# Recessive Antimorphic Alleles Overcome Functionally Redundant Loci to Reveal *TSO1* Function in *Arabidopsis* Flowers and Meristems

**DOI:** 10.1371/journal.pgen.1002352

**Published:** 2011-11-03

**Authors:** Paja Sijacic, Wanpeng Wang, Zhongchi Liu

**Affiliations:** 1 Department of Cell Biology and Molecular Genetics, University of Maryland, College Park, Maryland, United States of America; 2 Plant Science Graduate Program, Department of Plant Science and Landscape Architecture, University of Maryland, College Park, Maryland, United States of America; Peking University, China

## Abstract

*Arabidopsis TSO1* encodes a protein with conserved CXC domains known to bind DNA and is homologous to animal proteins that function in chromatin complexes. *tso1* mutants fall into two classes due to their distinct phenotypes. Class I, represented by two different missense mutations in the CXC domain, leads to failure in floral organ development, sterility, and fasciated inflorescence meristems. Class II, represented by a nonsense mutation and a T-DNA insertion line, develops wild-type–like flowers and inflorescences but shows severely reduced fertility. The phenotypic variability of *tso1* alleles presents challenges in determining the true function of *TSO1*. In this study, we use artificial microRNA, double mutant analysis, and bimolecular fluorescence complementation assay to investigate the molecular basis underlying these two distinct classes of phenotypes. We show that the class I mutants could be converted into class II by artificial microRNA knockdown of the *tso1* mutant transcript, suggesting that class I alleles produce antimorphic mutant proteins that interfere with functionally redundant loci. We identified one such redundant factor coded by the closely related *TSO1* homolog *SOL2*. We show that the class I phenotype can be mimicked by knocking out both *TSO1* and its homolog *SOL2* in double mutants. Such antimorphic alleles targeting redundant factors are likely prevalent in *Arabidopsis* and maybe common in organisms with many sets of paralogous genes such as human. Our data challenge the conventional view that recessive alleles are always hypomorphic or null and that antimorphic alleles are always dominant. This study shows that recessive alleles can also be antimorphic and can produce a phenotype more severe than null by interfering with the function of related loci. This finding adds a new paradigm to classical genetic concepts, with important implications for future genetic studies both in basic research as well as in agriculture and medicine.

## Author Summary

Much of our current genetic concepts and terms came from early pioneering work in *Drosophila melanogaster*, which has a relatively simple genome with reduced gene sets. One noted example is the term antimorph or dominant-negative, which describes mutant proteins that antagonize the corresponding wild-type proteins in a dominant fashion. In the process of characterizing *Arabidopsis thaliana tso1* mutants, we discovered a novel genetic phenomenon “recessive antimorphism,” where certain recessive and missense mutations interfere with functionally redundant genes in the genome to reveal a broader range of phenotypes than the corresponding loss-of-function or null alleles. Our work indicates a rarely noted strength of *Arabidopsis* as a genetic model for studying species with complex genome architecture, including humans that possess significant chromosome segmental or genome duplications and increased gene copy numbers. It adds a new paradigm to classical genetic concepts with important implications for modern genetics in both medicine and agriculture.

## Introduction

During the transition from vegetative to reproductive phase all flowering plants develop flowers from stem cells at the shoot apex, called the inflorescence meristem (IM). In *Arabidopsis thaliana*, the IM gives rise to indeterminate number of floral meristems (FM). Each FM develops and subsequently differentiates into a flower with four distinct types of floral organs. Much has been learned about how the four floral organ types are specified by the four classes of floral homeotic genes [1], [2]. However, very little is known about how each floral organ grows and differentiates into its final shape, size, and morphology. This is partly owing to difficulties in identifying and analyzing mutants that fail to grow and differentiate, as their phenotypes may not be as distinct as floral homeotic mutants.

**Figure 1:**
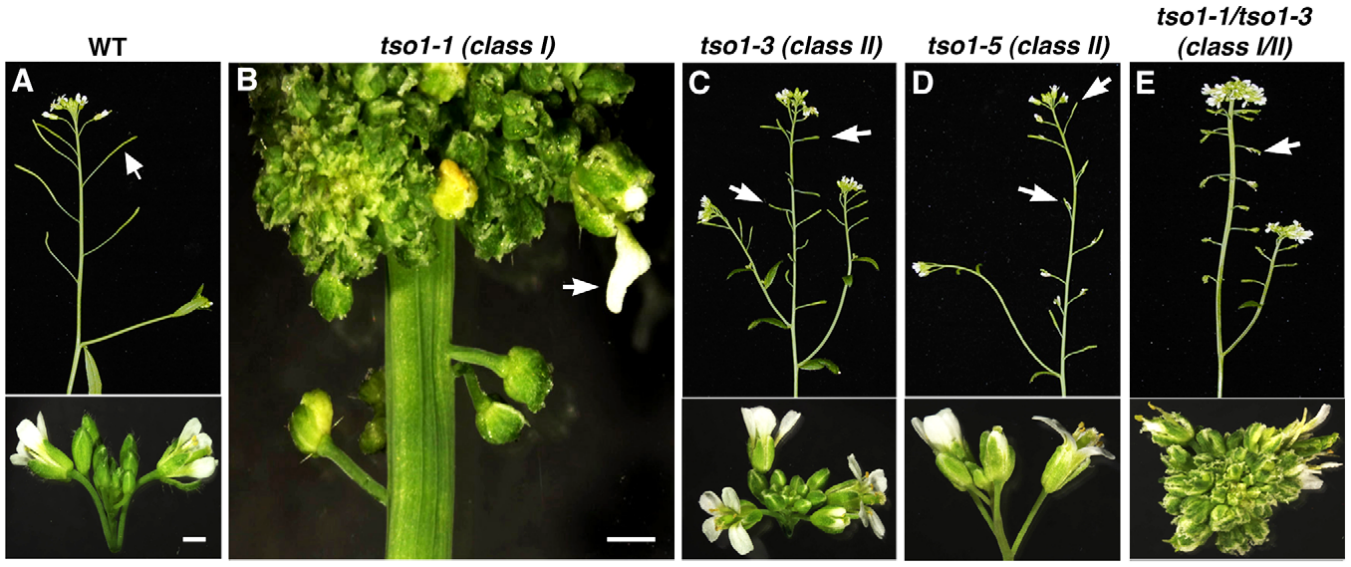
Phenotypic classes of *tso1* alleles. (A) A wild type (WT) plant with fertile siliques indicated by an arrow (top panel) and a normal inflorescence (bottom). (B) A *tso1-1* mutant inflorescence with an extremely fasciated meristem and undifferentiated flowers. Petal-like floral organ, occasionally seen in *tso1-1* flowers, is indicated by an arrow. (C) A *tso1-3* plant showing small siliques (arrows in top panel) but normal inflorescence and wild type-like flowers (bottom). (D) A *tso1-5* mutant plant showing similar phenotypes as *tso1-3* in (C). (E) A *tso1-1/tso1-3* transheterozygous plant showing a fasciated inflorescence (bottom) and a lack of silique (an arrow, top panel). Bars in A and B: 500 µm. C, D, and E are at the same magnification as A.

*Arabidopsis tso1-1* appears to belong to this second class of flower mutants, as *tso1-1* mutants fail to develop differentiated floral organs [3], [4]. Besides abnormal sepals, almost all other floral organs of *tso1-1* flowers are missing and are replaced by a mass of callus-like undifferentiated tissues (Figure 1B). Rarely, *tso1-1* flowers develop rudimentary floral organs, including petal-like structures (Figure1B) and unfused carpels. Since *tso1-1* mutant plants do not develop normal reproductive organs, plants are completely sterile. In addition to the floral organ differentiation defects, inflorescence meristems of *tso1-1* mutants are often enlarged and fasciated, splitting from one into several inflorescences (Figure 1B and [3], [4]). Although *TSO1* mRNA is detected in all *Arabidopsis* tissues *tso1-1* phenotypes are largely flower-specific.

Using map-based cloning, we and others showed that the *TSO1* gene (At3g22780) encodes a nuclear protein with two tandem cysteine-rich (CXC) repeats connected by a conserved intervening hinge region [3], [5]. Eight *TSO1* homologs (CXC-Hinge-CXC or CHC genes) have been described in *Arabidopsis* and can be grouped into two different types [3], [4], [6]. *TSO1* belongs to type I, together with two closely related homologs At3g22760 and At4g14770, which were also named as *SOL1* and *SOL2*, respectively [5], and a fourth member At3g04850. Type II homologs, which are phylogenetically more distant from type I, include four genes, At4g29000, At2g20110, At5g25790, and At3g16160. Among the type I CHC proteins, *TSO1* and *SOL2* show highly similar expression patterns throughout the plant except in pollen and carpel tissues, where *SOL2* is absent or expressed at a very low level [6]. On the other hand, *SOL1* is predominantly expressed in all stages of pollen development. *TSO1* transcript was also found in pollen development, but is limited to uninucleate microspores and bicellular pollen (not tricellular and mature pollen) [6]. The expression of the fourth member of the type I CHC proteins could not be detected and was suspected to encode a pseudogene.

The CHC proteins are absent in prokaryotes but present in all eukaryotes except fungi [6]. A CHC domain-containing protein was shown to bind DNA in soybean [7]. Also, CHC binds zinc ions and may define a novel zinc-finger domain [6]. The mammalian CHC protein, TESMIN, was originally identified in testes, but subsequently also detected in ovary development [8]–[10]. In *Drosophila melanogaster*, there are two CHC genes, *Mip120* (*myb-interacting protein 120*) and *Tombola,* whose gene products function in two paralogous chromatin complexes [11]–[14]. The dREAM complex contains the Mip120 and was found to regulate cell cycle and cell differentiation [14]–[16]. The tMAC complex contains Tombola and regulates testis-specific programs [13]. The *Caenorhabditis elegans* CHC protein LIN-54, a component of the orthologous DRM complex, was recently shown to recognize and bind a hybrid E2F/DP and LIN-54 consensus motif and help recruit DRM to promoters of genes involved in cell cycle, development, and reproduction [17]. Blast searches identified plant homologs of almost all dREAM chromatin complex components, suggesting the possibility of a plant dREAM-like complex, whose activity may depend on TSO1.

Several different *tso1* alleles have been previously described, all of which are recessive. The strongest allele is *tso1-1* caused by a missense mutation in the second CXC repeat, replacing one of the highly conserved cysteines by a tyrosine [3]. *tso1-2* allele, a result of replacing another conserved cysteine by a tyrosine in the first CXC repeat [3], caused a similar phenotype as *tso1-1*. In contrast, *tso1-3* is a nonsense mutation that causes premature protein termination after the first CXC domain [3], [5]. However, *tso1-3* phenotype is weak and differs significantly from *tso1-1* and *tso1-2*. *tso1-3* mutant plants develop normal flowers and do not exhibit meristem fasciation (compare Figure 1B with Figure 1C). The only defect is its severely reduced fertility as shown by the formation of very short siliques (seed pods) (Figure 1C and [5]). A fourth allele, *tso1-5*, was caused by a T-DNA insertion in the second CXC repeat, leading to undetectable levels of *TSO1* transcripts [6]. *tso1-5* is very similar to *tso1-3* phenotypically with morphologically wild type flowers but small siliques (Figure 1D and [6]). Therefore, *tso1* alleles can be grouped into two distinct classes. Class I includes *tso1-1* and *tso1-2* missense mutations that cause severe floral organ differentiation and meristem defects, and class II includes *tso1-3* and *tso1-5* loss-of-function mutations showing only reduced seed set.

1946 Nobel Prize winner H.J. Muller coined the terms amorph, hypomorph, hypermorph, antimorph and neomorph to indicate quantitative changes to the wild type characters based on his analyses of *Drosophila* mutants [18]. Today, “amorph” is often used interchangeably with “null”, hypomorph with “loss-of-function”, and antimorph with “dominant-negative”. Antimorphic (dominant-negative) mutant alleles, in a heterozygote state, antagonize the activity of corresponding wild type alleles to give a null-like phenotype and thus are thought to always act dominantly over wild type [18], [19].

The work reported here suggests that the *tso1* class I alleles are antimorphic alleles, which however act recessively to their wild type allele. Specifically, experiments were conducted to answer questions why there is such a dramatic phenotypic difference between the missense class I alleles (*tso1-1* and *tso1-2*) and the loss-of-function class II alleles (*tso1-3* and *tso1-5*) and what is the nature of the *tso1-1* and *tso1-2* missense mutations. Using gene knockdown (artificial microRNA), T-DNA insertions, double mutant analyses, and Bimolecular Fluorescent Complementation (BiFC) assay, we obtained genetic and molecular data indicating that class I are recessive antimorphic alleles, which lost their normal function but interfered with the activity of a TSO1 homolog SOL2. Our work provides important mechanistic insights into recessive antimorphism and has broad implications both for basic science and for medicine and agriculture.

## Results

### Artificial MicroRNA Knockdown of *TSO1* Suppressed *tso1-1* Flower Phenotype

One obvious question is what the *tso1* null allele is like. Since *tso1-1* and *tso1-2* (class I) exhibited stronger phenotypes, they could be null alleles. If the class I alleles were null, further reduction of *tso1* mutant transcripts should not cause any change in their phenotypes. Alternatively, *tso1-3* and *tso1-5* (class II) could be null alleles as they cause protein truncation and undetectable RNA transcript, respectively [3], [5], . Consequently, the class I (*tso1-1* and *tso1-2)* alleles, with a more severe mutant phenotype, are unlikely to be hypomorphic alleles. Instead, the class I alleles may act as recessive antimorphic alleles that not only lose *TSO1* function but also interfere with functionally redundant *TSO1* homologs, such as *SOL1* and *SOL2*. This would explain why these class I alleles possess a more severe phenotype than the class II (null) alleles. If this second scenario were true, further reduction of *tso1-1* transcripts in *tso1-1* plants may remove the antimorphic (interfering) effect of *tso1-1* and ameliorate the *tso1-1* phenotype.

**Figure 2:**
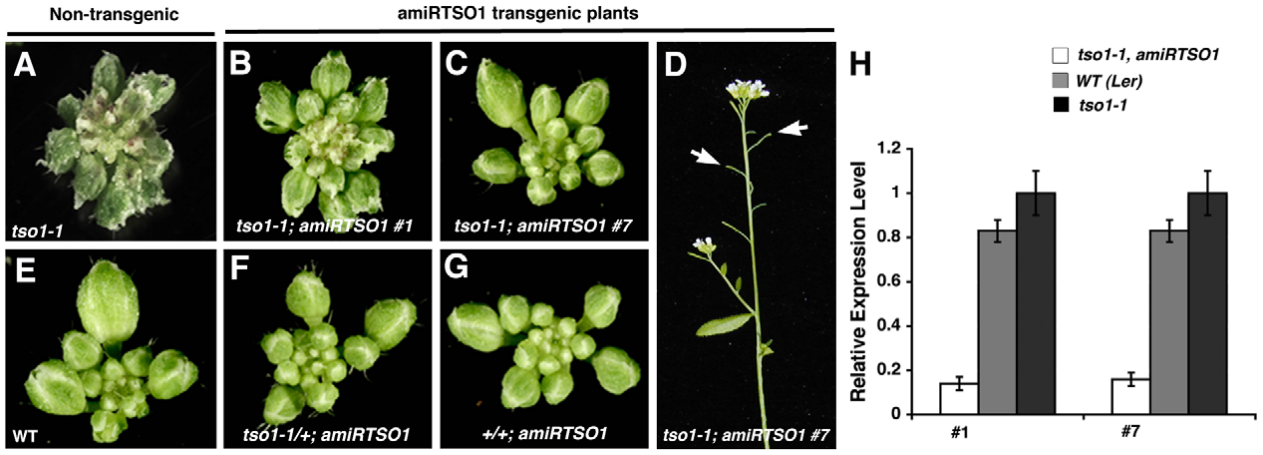
Artificial microRNA knockdown of *tso1-1* transcripts suppresses *tso1-1* phenotype. (A) A *tso1-1* mutant inflorescence showing flower buds with jagged sepals. (B) An inflorescence of *tso1-1; amiRTSO1* line #1 showing suppressed floral phenotype. (C) An inflorescence of *tso1-1; amiRTSO1* line #7 with an even more suppressed floral phenotype. (D) Side view of a *tso1-1; amiRTSO1* line #7 plant showing short and seedless siliques (arrows). (E) A wild type (L*er*) inflorescence. (F) A *tso1-1/+; amiRTSO1* inflorescence. (G) A +/+; *amiRTSO1* inflorescence. (H) Real time RT-PCR analysis of *tso1-1* transcript levels in *tso1-1; amiRTSO1* (white bars) or *tso1-1* (black bars) plants. The *TSO1* transcript level in wild type (L*er*, grey bars) is also shown. The relative level of *tso1-1* transcripts in *tso1-1* plants was designated as 1. Standard deviation was calculated based on three technical replicates.

To test the above alternative hypotheses, an artificial microRNA was used to knock down *tso1-1* mutant transcripts in *tso1-1* mutants. This artificial microRNA construct, named *2044amiRTSO1,* was designed to specifically target the 3′ end of the *TSO1* gene (see Materials and Methods). Wild type plants were transformed with the construct to yield 63 first generation (T1) *2044amiRTSO1(WT)* transgenic lines, none of which showed any phenotype (Figure 2G). Since *tso1-1* homozygous plants are sterile, *tso1-1/+* heterozygous plants were transformed with the *2044amiRTSO1* construct to yield 43 T1 transgenic lines. Four such lines were identified to be *tso1-1/+; amiRTSO1* by genotyping, and all of them showed wild type phenotype (Figure 2F). On the other hand, five plants genotyped as *tso1-1; amiRTSO1* exhibited inflorescence and flower phenotypes that were much milder than *tso1-1* single mutants (compare Figure 2A with Figure 2B-2C, and Figure 1B with Figure 2D), indicative of a suppression of the *tso1-1* phenotype by the *amiRTSO1*. To confirm that the observed phenotypic suppression in the *tso1-1; amiRTSO1* plants was due to a reduction of *tso1-1* transcripts, real-time RT-PCR was performed on two *tso1-1; amiRTSO1* T1 transgenic lines, #1 and #7 (Figure 2H). The level of *tso1-1* transcripts in both lines was reduced to about 15% of the untransformed *tso1-1* level, suggesting that the reduction of *tso1-1* mutant gene products in *tso1-1; amiRTSO1* plants may underlie the phenotypic suppression.

This result provides strong support for the second scenario that *tso1-1*, as well as other class I alleles, are likely recessive antimorphic alleles, while class II alleles (*tso1-3* or *tso1-5*) are loss-of-function or near complete loss-of-function (near-null) alleles. The significant reduction of *tso1-1* mutant gene products in *tso1-1; amiRTSO1* plants removed the interfering effects of *tso1-1* on potentially redundant factors.

### Class II Alleles Cause Severe Fertility Defects

**Figure 3:**
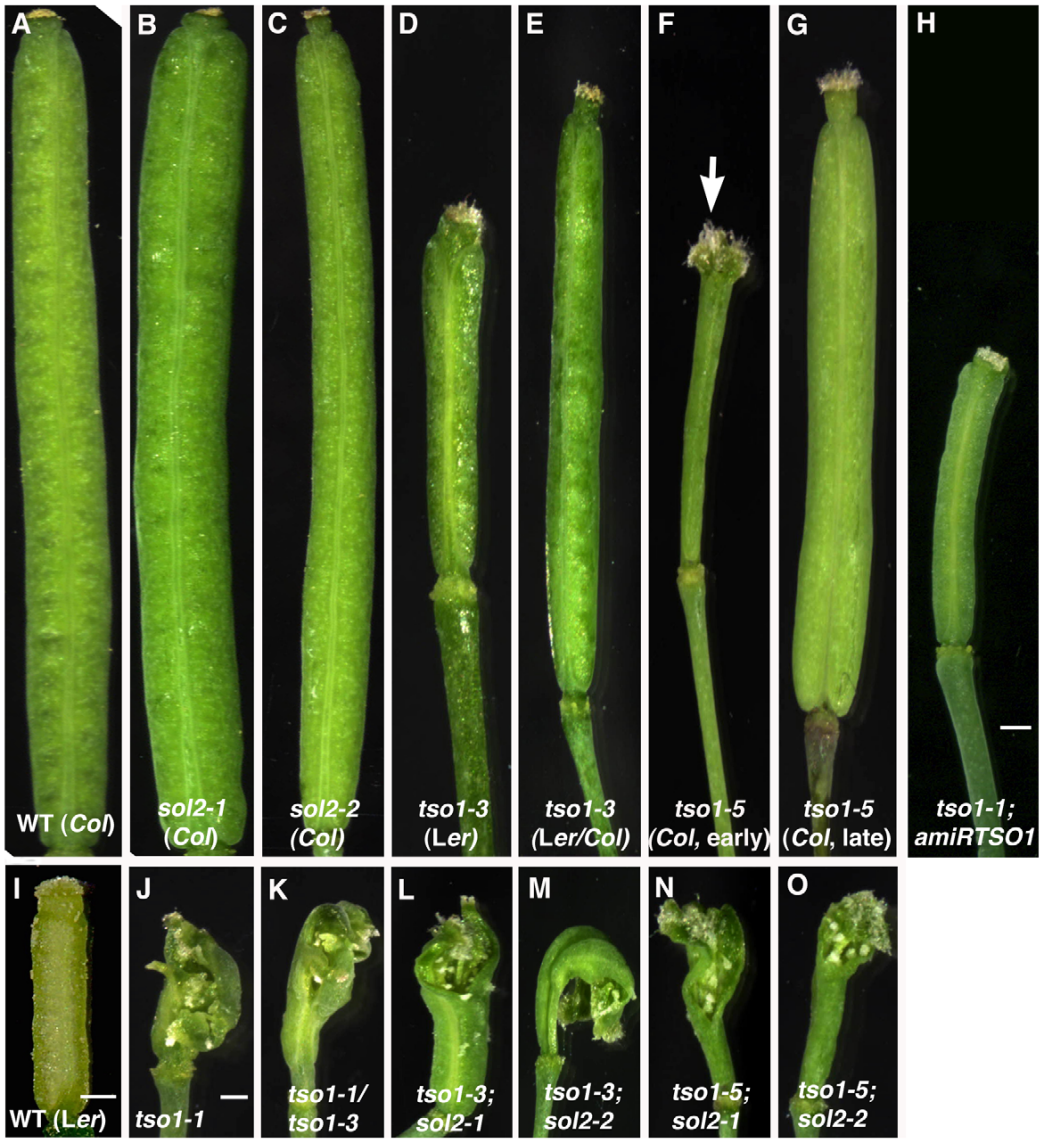
*tso1-3; sol2* or *tso1-5; sol2* double mutants exhibit fertility defects similar to those of *tso1-1* single mutants. (A) A silique (seed pod) of wild type (WT). (B) A silique of *sol2-1*. (C) A silique of *sol2-2*. (D) A *tso1-3* silique. *tso1-3* is in L*er* background. (E) A *tso1-3* silique in mixed L*er*/*Col* background. (F) A gynoecium (a female reproductive organ) of *tso1-5* that failed to develop into a silique. Note the abnormal gynoecium apical area (arrow). (G) A silique of *tso1-5* (*Col*) formed later during plant development. (H) A silique of *tso1-1; amiRTSO1*. (I) A WT gynoecium consists of two fused carpels at the time of anthesis. (J–O) Absence of silique formation and the persistence of abnormal gynoecium in *tso1-1* (J), *tso1-1/tso1-3* (K), *tso1-3; sol2-1* (L), *tso1-3; sol2-2* (M), *tso1-5; sol2-1* (N), and *tso1-5; sol2-2* (O). Bars in H–J: 200 µm. All photos in A–O (except I) are at the same magnification. Photos of A–C were compressed vertically by 30% to fit the figure height.

Although the *tso1-1; amiRTSO1* plants described above were able to clear the antimorphic *tso1-1,* they still lack wild type *TSO1* and thus resemble class II mutant plants. Specifically, *tso1-1; amiRTSO1* plants formed extremely short siliques and were completely sterile despite their nearly normal floral and meristem development (Figure 2D, Figure 3H). Therefore, *amiRTSO1* was able to convert a class I allele into a class II allele, and class II alleles represent severe loss-of-function or near-null alleles.

It is important to note that the severe fertility defects observed for both class I and class II alleles have rather distinct causes. The complete sterility of class I alleles are due to failure of proper reproductive floral organ formation. Figure 3J and 3K showed *tso1-1* and *tso1-1/tso1-3* mutant gynoecium (female reproductive organ) consisting of unfused and abnormally formed carpels that expose rudimentary ovules at the apex. In contrast, the gynoecia of class II alleles are wild type-like, shown by the two perfectly fused carpels (Figure 3D–3H). The reduced seed sets in class II mutants apparently result from defects in male and female gametes. The size of the silique (seed pod) positively correlates with the number of seeds inside. *tso1-1; amiRTSO1* has the smallest silique (Figure 2D, Figure 3H) and is completely sterile. This is followed by *tso1-3* with 0-1 viable seed per small silique (Figure 3D), and finally by *tso1-5* with 1–5 viable seeds per silique (Figure 3F–3G). *tso1-5* plants at first appeared to have severe fertility defects (Figure 3F). However, siliques that developed later from the same shoot were longer and had more seeds (Figure 3G).

The distinct phenotypes between class I and class II alleles are not caused by different ecotype backgrounds as the class I alleles, *tso1-1* and *tso1-2*, and the class II allele *tso1-3* are all in the L*er* background. However within class II, *tso1-3* (L*er*) is less fertile than *tso1-5* (*Col*) (Figure 3D and 3G), even though both alleles cause TSO1 protein truncation after the first CXC domain. By crossing *tso1-3* into the *Col* background, the fertility of *tso1-3* became similar to *tso1-5* (Figure 3E and 3G). Therefore, the extent of infertility of class II mutants could be influenced by the ecotype.

### The *tso1-1* Antimorphic Allele Is Intrinsically Recessive to the *TSO1* Wild-Type Allele

Antimorphic alleles, also termed “dominant-negative” alleles, usually interfere with the function of their wild type alleles and are defined as dominant alleles [18], [19]. *tso1-1* appears to violate this rule as it is recessive to its wild type allele, but at the same time antimorphic in nature. One hypothesis is that *tso1-1* may act in a dosage-dependent manner, being recessive when *tso1-1* equals wild type in dosage. A higher *tso1-1* dosage may overcome *TSO1* wild type allele and cause a mutant phenotype. An alternative hypothesis is that *tso1-1* is not antimorphic to its wild type allele (thus recessive to the wild type) but rather antimorphic to other *TSO1* redundant factors. To test these hypotheses, 35S::*tso1-1* (full-length *tso1-1* cDNA driven by the strong 35S promoter) was introduced into wild type (L*er*) plants. Out of 76 T1 transgenic lines, none showed any mutant phenotypes. In the T2 generation, where 25% of *35S::tso1-1* plants should become homozygous for the transgene, still none showed any mutant phenotype. The transcript level of *tso1-1* from four independent T2 *35S::tso1-1* transgenic lines was assayed by RT-PCR and shown to be at a higher level than the endogenous *TSO1* (Figure S1). These results suggest that the over-expressed *tso1-1* mutant gene product was unable to cause a mutant phenotype when wild type *TSO1* is present and that *tso1-1* is recessive to wild type *TSO1* irrespective of its dosage.

The above conclusion is further supported by the reciprocal experiment, where *TSO1* wild type cDNA was over-expressed in *tso1-1* plants under the control of the *35S* promoter. Specifically, *35S::TSO1-GFP* transgene was introduced into *tso1-1/+* plants. Through genotyping in T1 generation, four *tso1-1; 35S::TSO1-GFP* lines were identified. Three lines were completely rescued and are indistinguishable from wild type. The remaining one was not rescued probably due to positional effect of the transgene. Combined, our data suggest that *tso1-1* readily succumbs to *TSO1* wild type allele irrespective of its dosage to the wild type *TSO1* and that *tso1-1* only exerts its effect when wild type *TSO1* is absent.

In *tso1-1/tso1-3* (classI/classII) transheterozygotes, the amount of tso1-1 mutant protein is at 50% of the *tso1-1/tso1-1* plants and the abnormal floral organ, fertility and meristem phenotypes are similar but milder than *tso1-1* homozygotes (compare Figure 1B with 1E, and Figure 3J with 3K), suggesting that *tso1-1* acts in a dosage-dependent fashion to interfere with some unknown factor(s) to yield the floral and meristem phenotype.

### T-DNA Insertions in *SOL1* and *SOL2* Genes Caused No Phenotypic Defects

Possible redundant factors that *tso1-1* interferes with could be the two most closely related *TSO1* homologs, *SOL1* and *SOL2*. *SOL2* is the most likely candidate as it is expressed in a highly similar pattern to *TSO1* with the only exception of pollen and carpel tissues, where *SOL2* is absent [6]. It would explain why *tso1* class II alleles never exhibit any defects in flowers and inflorescence meristems, as defects in these tissues are “masked” by the redundantly acting *SOL2*.

**Figure 4:**
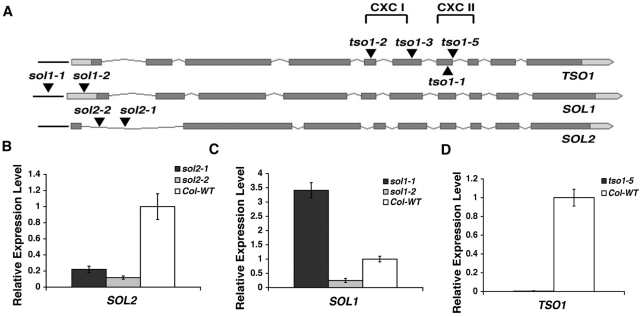
Analyses of T-DNA insertion lines in *TSO1*, *SOL1*, and *SOL2*. (A) Positions of mutations in *TSO1, SOL1,* and *SOL2.* Full-length genomic sequences, including promoters and 5′ and 3′ UTRs, are shown. Dark boxes represent exons. Light boxes indicate the UTRs. Grey thin lines represent introns. Black lines represent promoters. Black-filled triangles mark the position of T-DNA insertions or *tso1* alleles. *tso1-5* (*Salk_102956*) T-DNA is inserted in the seventh intron very close to the exon-intron boundary. *sol1-1* (*Salk_007957*) is inserted in the *SOL1* promoter. *sol1-2* (*Salk 013686*) is inserted in the *SOL1* 5′UTR. *sol2-1* (*Sail 78_A12*) and *sol2-2* (*Salk_021952*) are both inserted in the first intron of *SOL2*. *tso1-2* missense allele mutates a conserved cysteine in the first CXC domain. *tso1-3* is a nonsense mutation in the hinge region between the two CXC domains. *tso1-1* mutates a conserved cysteine located in the second CXC domain. (B–D) Real time RT-PCR analysis of *SOL2, SOL1,* and *TSO1*. Standard deviations were calculated based on two biological replicates, each with three technical replicates. The wild type level for each gene was assigned as value 1.

**Figure 5:**
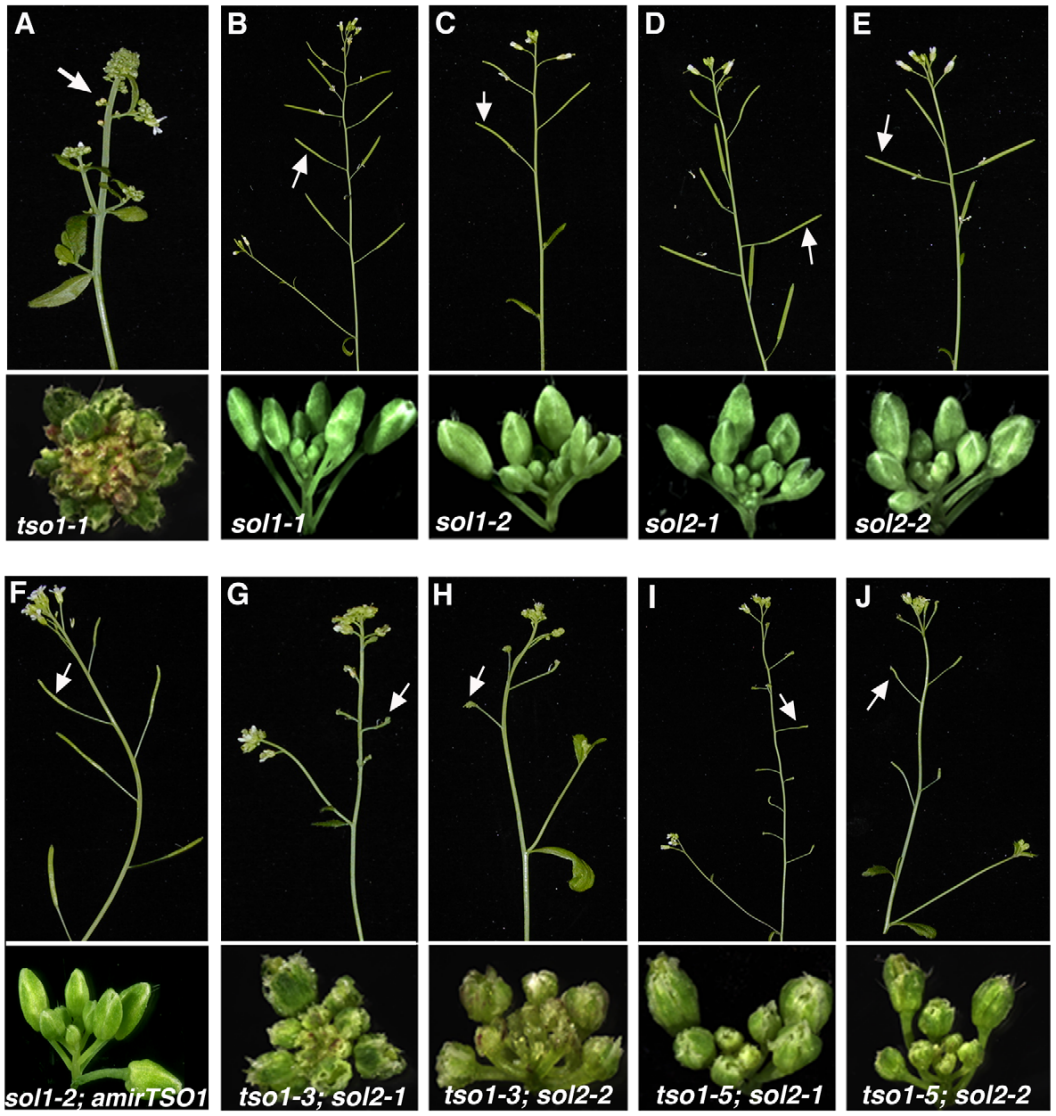
*sol2*, but not *sol1*, showed synergistic genetic interactions with *tso1* class II alleles. Each panel consists of the top portion showing fertility phenotypes and bottom portion showing flower and inflorescence phenotypes. (A) *tso1-1*. (B–E) Fully fertile siliques and morphologically normal inflorescences developed in *sol1-1* (B), *so1-2* (C), *sol2-1* (D), and *sol2-2* (E) single mutants. (F) A *sol1-2; amiRTSO1* inflorescence showing wild type phenotype. (G–J) *tso1; sol2* double mutant plants showing an absence of silique and abnormal inflorescences of *tso1-3; sol2-1* (G), *tso1-3; sol2-2* (H), *tso1-5; sol2-1* (I), and *tso1-5; sol2-2* (J).

First, we characterized single mutants of *SOL1* and *SOL2*. Two different T-DNA insertion lines for each gene were obtained from the ABRC stock center (Figure 4A). Real-time RT-PCR with gene specific primers demonstrated that *sol2* alleles have a reduced *SOL2* expression at about 20% of the wild type level (Figure 4B), and thus they may be loss-of-function alleles. On the other hand, two different *sol1* alleles, *sol1-1* and *sol1-2*, showed opposite effects on *SOL1* expression (Figure 4C). In *sol1-1*, *SOL1* expression was increased about three fold when compared to the wild type, most likely as a result of the T-DNA insertion in the *SOL1* promoter (Figure 4A). In contrast, *SOL1* expression was reduced to about 30% of the wild type level in *sol1-2*, and could represent a loss-of-function allele (Figure 4C). Although the expressions of *SOL1* and *SOL2* genes in corresponding T-DNA lines were dramatically changed, the mutant plants look indistinguishable from the wild type (*Col*) plant (Figure 5B–5E).

We also characterized *tso1-5*, a T-DNA line inserted in the seventh intron of *TSO1* gene near the exon-intron boundary (Figure 4A). Consistent with the previous report [6], real-time RT-PCR analysis showed undetectable levels of *TSO1* mRNA in *tso1-5* (Figure 4D).

### *sol2* But Not *sol1* Showed Synergistic Genetic Interactions with *tso1* Class II Alleles

To determine if *SOL1*, *SOL2*, or both encode the redundant factor(s), we aimed to construct double mutants between *tso1* class II alleles and *sol1* or *sol2* loss-of-function alleles. Since *SOL1* (At3g22760) and *TSO1* (At3g22780) are closely linked on chromosome 3 (only 1545 bp apart and with one gene, At3g22770, in between), we couldn't construct double *sol1 tso1* mutants. Instead, we knocked down *TSO1* by crossing the *amiRTSO1* into *sol1-2,* which has reduced *SOL1* transcripts due to a T-DNA insertion at the 5′UTR (Figure 4A, 4C). The *sol1-2; amiRTSO1* plants showed a wild type phenotype (Figure 5F) even though the *amiRTSO1* caused a significant reduction of *TSO1* (at about 11% of the wild type level) in the *sol1-2; amiRTSO1* plant (Figure S2). This result suggests that *SOL1* is unlikely a redundant factor of *TSO1*.

To test if *SOL2* encodes a redundant factor for *TSO1*, we constructed *sol2; tso1* double mutants by crossing *sol2* loss-of-function alleles into the *tso1* class II loss-of-function alleles, *tso1-3* and *tso1-5*. If *tso1-1* acts to interfere with the function of *SOL2* then *sol2; tso1-3* or *sol2; tso1-5* double mutants should resemble *tso1-1* mutants (Figure 5A). Genotyping identified *tso1-3; sol2-1* and *tso1-3; sol2-2* double mutants in F2, which showed severe morphological abnormalities in floral organs similar to those of *tso1-1* (compare Figure 5A with Figure 5G–5H). The *tso1-3; sol2* double mutants are completely sterile bearing no silique nor seeds. Instead, gynoecium with unfused carpels was formed (compare Figure 3B–3E, 3J–3K with Figure 3L–3M). Similar fertility and floral morphology defects were also observed in *tso1-5; sol2-1* and *tso1-5; sol2-2* double mutant plants (Figure 5I–5J, compare Figure 3B–3C, 3F–3G, 3J–3K with Figure 3N–3O), which were slightly weaker than those of *tso1-3; sol2* double mutants.

Nevertheless, the *tso1-3; sol2* and *tso1-5; sol2* double mutants rarely showed meristem fasciation defects, which are typically seen in *tso1* class I alleles. It is likely that additional redundant factor(s) may need to be knocked down in *tso1-3; sol2* mutants to fully manifest the *tso1-1* phenotype. Alternatively, the *sol2* alleles used in the study may still retain some residual function, as 20% of the *SOL2* transcript is still present in *sol2* mutants (Figure 4B).

### Biomolecular Fluorescence Complementation (BiFC) Assay Detected Direct Interaction between tso1-1 and SOL2

**Figure 6:**
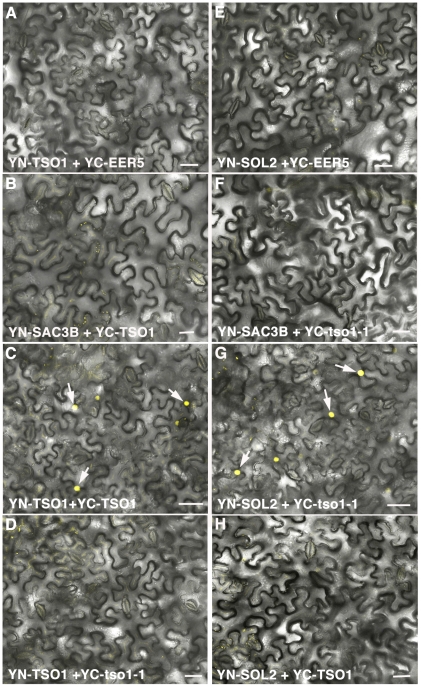
BiFC analyses showing TSO1 to TSO1 as well as tso1-1 to SOL2 interactions. Interactions were detected by YFP reconstitution between the YN and YC fusion proteins, leading to yellow fluorescence shown by single confocal section images overlaid with Nomarsky differential interference contrast (DIC) images. YC-EER5 and YN-SAC3B nuclear proteins serve as negative controls as they function in unrelated processes from TSO1 or SOL2 [39]. Arrows point to nuclei expressing YFP fluorescence. Scale bars represent 50 µm. (A–B) Negative control combination of YN-TSO1 with YC-EER5 (A), or YN-SAC3B with YC-TSO1 (B). (C) Positive interaction between YN-TSO1 and YC-TSO1 indicated by fluorescent nuclei (arrows). (D) An absence of interaction between YN-TSO1 and YC-tso1-1 indicated by an absence of fluorescent signals. (E–F) Negative control combination of YN-SOL2 with YC-EER5 (E), or YN-SAC3B with YC-tso1-1 (F). (G) *in vivo* interaction between YN-SOL2 and YC-tso1-1 shown by fluorescent nuclei (arrows). (H) An absence of interaction between YN-SOL2 and YC-TSO1.

Our genetic data above strongly suggest that *TSO1* and *SOL2* act redundantly during flower and, less so, meristem development. Only when the function of both genes is compromised, either through antimorphic *tso1-1* or by double knockdown, the class I phenotype can be revealed. To further investigate the molecular mechanisms underlying this genetic interaction, we tested direct physical interactions among TSO1, tso1-1, and SOL2 using BiFC. *TSO1, tso1-1,* and *SOL2* cDNAs were fused in frame to the YFP N-terminal (YN) or the YFP C-terminal (YC) fragments. Pairs of YN and YC fusion constructs were co-infiltrated into the leaf epidermis of *Nicotiana benthamiana*. Direct interactions between the YN and YC fusion proteins can be detected by the YFP reconstitution and yellow fluorescence. YC-EER5 and YN-SAC3B are nuclear proteins, serving as negative controls in combination with test proteins. Wild type TSO1 was able to interact with itself in nuclei (compare Figure 6A–6B with Figure 6C) but was unable to interact with either SOL2 (Figure 6H) or tso1-1 (Figure 6D). This suggests that TSO1 may act independently of SOL2, for example, by acting in a different complex from SOL2. The absence of interaction between TSO1 and tso1-1 (Figure 6D) excludes the possibility of tso1-1 interfering with TSO1 through direct binding. Interestingly, strong nuclear YFP fluorescence was observed when YN-SOL2 and YC-tso1-1 were co-infiltrated (compare Figure 6E–6F with Figure 6G). This suggests a possible mechanism of tso1-1 sequestering or blocking SOL2 from performing SOL2 normal function in nucleus.

## Discussion

We observed and characterized two classes of *tso1* alleles that exhibit dramatically different phenotypes. While the class I *tso1* alleles, exemplified by the missense mutations of conserved cysteine residues in the CXC domain, develop abnormal floral organs and exhibit meristem fasciation, the class II *tso1* alleles, represented by the *tso1* nonsense allele and the T-DNA insertion allele, do not show any such defects in floral organ morphology or meristem fasciation, but rather they develop small siliques with reduced seed set. We showed that the class I phenotype can be suppressed and converted into the class II phenotype by artificial microRNA knockdown of the *tso1* mutant transcript in class I mutants. This suggests that the class I alleles yielded antimorphic mutant products that were removed by the artificial microRNA and that the class II *tso1* alleles are null or near-null alleles.

### Antimorphic Alleles May Target Related Loci

Classical antimorphic alleles are only known to interfere with the wild type function at the same locus and are dominant over wild type [18], [19]. Here we show that an antimorphic allele can also interfere with the function of different loci with redundant functions. Such antimorphic alleles could serve as a powerful tool in the identification of gene function coded by functionally redundant gene families. We proposed that the antimorphic *tso1-1* interferes with SOL2 and possibly other TSO1 family members. By removing functionally redundant factors, the *tso1-1* antimorphic allele reveals a broader spectrum of TSO1 functions that are otherwise masked by the presence of redundant genes.

Our finding of *SOL2* instead of *SOL1* encoding the redundant factor is consistent with the highly similar tissue expression patterns between *TSO1* and *SOL2* throughout the plant except that *SOL2* is not expressed or is expressed at a low level in pollen and ovule. Therefore, *tso1* loss-of-function or null alleles only exhibit fertility defects due to an absence of *SOL2* expression during the development of male and female gametes. On the other hand, *SOL1* is predominantly expressed in all stages of pollen development, yet *tso1* class II alleles still exhibit reduced male fertility supporting a non-redundant function between *TSO1* and *SOL1*.

### A Model on the Molecular Mechanism of *tso1-1* Antimorphism

Currently, little is known about how TSO1 proteins function to regulate floral organ differentiation, meristem regulation, and gametophyte development. Based on the study of CHC-containing proteins in animal systems, TSO1 may function in a dREAM-like chromatin complex. Another important class of chromatin regulators, the Enhancer of zeste E(z) polycomb group proteins, contain two tandem CXC domains but lack the intervening hinge domain. Missense mutations of the conserved cysteine residue of CXC in the *Drosophila* E(z) proteins prevented the E(z)-containing complex (PRC2) from binding to polytene chromosomes [20], suggesting that one way tso1-1 could affect the chromatin complex is to impair its ability to bind DNA targets.

**Figure 7:**
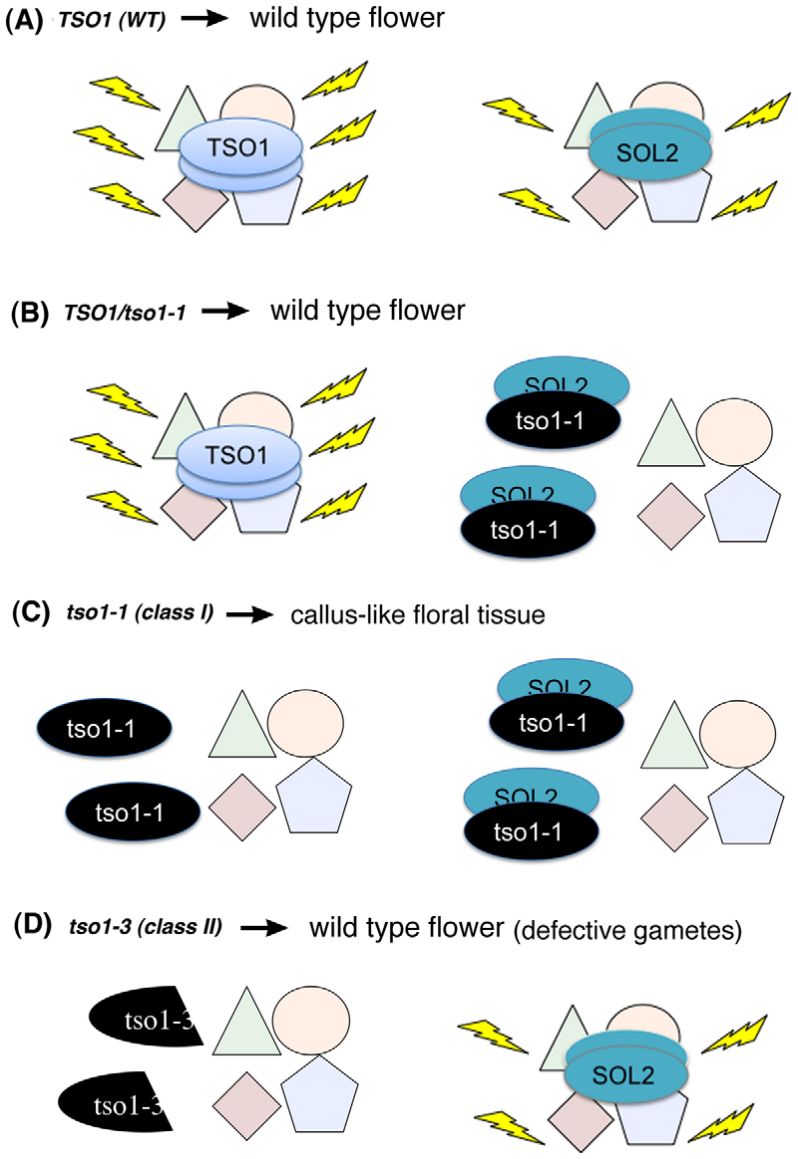
A proposed model on the molecular mechanism of *tso1-1* antimorphism in the context of flower development. (A) In wild type (WT), TSO1 and SOL2 function as essential components of two independent, yet functionally redundant, chromatin complexes. Yellow flashes indicate functional complexes. (B) In *tso1-1/TSO1* heterozygous plants, TSO1, at half of the wild type amount, is sufficient to confer wild type phenotype even in the presence of tso1-1, which completely or partially disables SOL2. (C) In *tso1-1/tso1-1* (class I) mutants, both TSO1 and SOL2 are nonfunctional due to an absence of wild type TSO1 and the inhibition of SOL2 by tso1-1. (D) In *tso1* class II mutants, such as *tso1-3* or *tso1-5*, SOL2 is functional and compensates for a lack of TSO1, leading to the development of normal flowers.

To gain insights into the molecular mechanism underlying class I *tso1* antimorphism, we tested direct physical interaction among TSO1, tso1-1, and SOL2 using BiFC (Figure 6). While wild type TSO1 could interact with itself but not with SOL2, the antimorphic tso1-1 could no longer interact with wild type TSO1 but could interact strongly with SOL2. This suggests that tso1-1 may interfere with SOL2 function by direct binding and then disabling of SOL2. The model illustrated in Figure 7 provides one of several possible mechanisms, explaining different phenotypic outcomes in different *tso1* genotypes. This model is proposed in the context of floral organ development where both TSO1 and SOL2 provide similar and redundant function in wild type (Figure 7A). In tso1-1/TSO1 heterozygotes (Figure 7B), a lack of physical interaction between tso1-1 and TSO1 excludes the possibility of tso1-1 interfering with TSO1 through direct binding. The presence of TSO1 wild type product is sufficient for the development of wild type flowers even when tso1-1 disables the SOL2. This is supported by the genetic dominance of wild type *TSO1* over *tso1-1* shown by the wild type phenotype of *tso1-1/+,* or *35S::tso1-1* (in wild type background), or *35S::TSO1-GFP (*in *tso1-1* background*)* plants. In *tso1-1* (class I) plants (Figure 7C), both TSO1 and SOL2 complexes are nonfunctional due to an absence of wild type TSO1 and the inhibition of SOL2 by tso1-1. In *tso1-3* (class II) mutants (Figure 7D), although wild type TSO1 is absent, the SOL2 complex provides sufficient function for normal flower development.

### Recessive Antimorphic Alleles Are Likely Common in *Arabidopsis*

One might ask how common these recessive antimorphic alleles exist. Through our own work, as well as brief surveys of *Arabidopsis* literature, we found several cases similar to *tso1-1*. Two recessive missense alleles of *BELLRINGER (BLR)*, *blr-4* and *blr-5,* cause conserved amino acid change in the homeodomain and exhibit a phenotype of terminal carpelloid flowers [21] rarely observed in loss-of-function or null alleles [22]–[24]. blr-4 and blr-5 were proposed to interfere with other family members harboring redundant functions [21]. In a second case, three *Arabidopsis* genes encode the small subunit of the *Ribonucleotide Reductase (RNR). tso2-1* (L*er*) is a recessive missense mutation in one of these *RNR* genes, causing a strong flower and inflorescence phenotype [25]. In contrast, *tso2-5*, a T-DNA insertion at the N-terminal end of the *TSO2* gene and thus a putative null, showed wild type phenotype (Wang and Liu, unpublished). In a third case, a recessive mutation of the *Arabidopsis CORONA (CNA)* gene, *cna-1*, located in a conserved domain of unknown function, showed a much stronger phenotype than a likely null allele, *cna-2* [26]. In the above examples, the recessive missense alleles may interfere with the function of redundant factors to cause a different phenotype or exhibit a stronger phenotype than the corresponding null. In addition to recessive antimorphic alleles, there are many examples of semi-dominant or dominant missense alleles that act to interfere with the function of redundant factors. The *clavatav1 (clv1)* missense mutations bear striking parallels to the *tso1* missense mutations [27]–[29]. The phenotypically medium to strong *clv1* alleles were all missense alleles, while the weak *clv1* alleles were all null or near-null. Co-suppression of *clv1* missense alleles led to weakened phenotypes closely resembling the *clv1* null [28]. The *clv1* missense alleles were thought to interfere with a *CLV1* homolog as well as with the wild type *CLV1*. In a second example, an unusual mutant allele of *APETALA 2 (AP2), I28*, exhibited a severe defect in shoot meristem development [30], which was observed in none of the previously characterized *ap2* mutants. *l28* causes a Glu to Lys change in the first AP2 domain that may be antimorphic by interfering with the function of a redundant factor, unmasking the function of *AP2* in shoot meristems [30]. The *Arabidopsis topless-1* (*tpl-1*) mutation transforms the shoot pole into a second root pole and *tpl-1* is a dominant-negative mutation that interferes with the function of multiple TPL-related proteins in embryo development [31]. These examples illustrate the advantage of using antimorphic alleles, irrespective recessive or dominant, to unveil the role of functionally redundant gene family members.

We propose that the distinction between “recessive” and “dominant” antimorphic alleles resides in whether the antimorphic allele interferes with its wild type allele. If it does, dominant or semi-dominant effect results. If it does not, as shown for *tso1-1*, recessive effect results. Whether “recessive” or “dominant”, the antimorphysm is not limited to interfering with its own locus but also with related loci.

### Broader Implications

Our findings have several important implications. First, null or near-null alleles, such as nonsense or T-DNA insertion alleles, are not necessarily always effective in revealing full gene functions when compared with missense mutations. Cautions should be exercised in making conclusions based on null alleles, especially those whose defective genes belong to gene families. While class I antimorphic alleles are able to reveal the range of TSO1 functions, the class II severe hypomorph or near-null alleles only reveal a subset of TSO1 function not complemented by the redundant factors. Second, antimorphic alleles can sometimes be recessive. In another word, not all antimorphic alleles are dominant or semi-dominant as defined in classical genetic analyses of *Drosophila* [18], [19]. Both dominant and recessive antimorphic alleles may interfere with the function of genes belonging to the same family. Third, our data challenge the conventional view that recessive alleles are always simple loss-of-function or null alleles. A different scenario illustrated in this study suggests that recessive alleles could also be antimorphic. Thus, alternative strategies aimed at eliminating rather than rescuing a genetic defect should be considered in ameliorating genetic abnormalities or diseases caused by recessive missense mutations.

Our work is potentially highly relevant to the study and interpretation of human genetic diseases. One example could be the wide spectrum of human diseases caused by mutations in the human A type lamin (LMNA) [32]. More than 10 different clinical syndromes including diseases of striated muscle, lipodystrophy syndromes, peripheral neuropathy, or accelerated aging are caused by various mutations in the LMNA gene. The striated muscle phenotype appears to be sensitive to reduced expression of LMNA and may represent hypomorphic alleles, while other symptoms might result from specific missense or splicing mutations that could lead to antimorphic LMNA proteins that interfere with LMNB or LMNC function. Our study reveals the strength of *Arabidopsis* as a genetic model whose gene number and genetic architecture are more appropriate for studying complex species like human, which is rich in low-copy repeats and paralogous segmental duplications (5%–10% of the human genome) [33]. Other models, such as *Drosophila* and *C. elegans*, have reduced gene sets, and thus reduced likelihood of discovering phenomena such as the recessive antimorphism discussed here.

## Materials and Methods

### Plant Growth, Mutant Strains, and Genetics

*Arabidopsis thaliana* plants were grown on Metromix soil (Griffin) under a 16 hour light-8 hour dark cycle at 20°C. *tso1-1* and *tso1-3* in Landsberg *electra* (L*er*) background were previously described [3]–[5], [34]. *tso1-1* and *tso1-3* genotyping was done by standard PCR using primers listed in the Table S1. Since *tso1-1* is 100% sterile, it is maintained as *tso1-1+/+sup-5* (L*er*/L*er*) heterozygote that was transformed with all constructs described.

The following T-DNA insertion lines (in *Columbia*; *Col* background) were obtained from ABRC stock center: Salk_102956 (*tso1-5,* At3g2278*0*), Salk_007957 (*sol1-1*, At3g 22760), Sail 742_H03 (*sol1-2*, At3g22760), Sail 78_A12 (*sol2-1*, At4g14770), and Salk_021952 (*sol2-2*, At4g14770). The genotyping of the T-DNA insertion lines was performed using standard PCR with primer pairs listed in Table S1. Conditions for standard PCR reaction were: 95°C for 3 minutes (min), followed by 35 cycles of 94°C for 30 seconds (s), 55°C for 30 s, 72°C for 75 s, and 72°C for 7 min.

### Gene Expression Studies

Total RNA was isolated from inflorescences of wild type, *tso1-1*, *sol1-1*, *sol1-2*, *sol2-1*, *sol2-2*, transgenic *2044 amiRTSO1 (tso1-1)*, *2044 amiRTSO1(Ler); sol1-2*, and *35S::tso1-1 (Ler)* plants using RNeasy Plant Mini Kit (Qiagen Inc, Valencia CA, USA). First-strand cDNA was synthesized from 1 µg of total RNA using QuantiSure^™^ First-strand cDNA Kit (Accugen Biosciences, Rockville MD, USA). 1 µl of 10X diluted cDNA was used as a template in real-time and RT-PCR analysis. iQ^™^ SYBR^®^ Green Supermix (Bio-Rad Laboratories, CA, USA) was used to set up real-time PCR reactions, which were run and analyzed on CFX96 Real-Time System (Bio-Rad Laboratories). Conditions for real-time PCR were as follows: 95°C for 3 min, followed by 40 cycles of 94°C for 15 s, 60°C for 15 s, 72°C for 30 s. Melting curve analysis was performed from 65°C to 95°C with increments of 0.5°C every 5 seconds. Gene specific primers and corresponding real-time PCR efficiencies for each primer pair are listed in Table S2. Primers used to test T-DNA lines were designed to detect transcripts 3′ of the insertion. The housekeeping gene *GLYCERALDEHYDE-3-PHOSPHATE DEHYDROGENASE C SUBUNIT 1* (*GAPC1*, At3g04120) was used as a reference gene in all real-time PCR reactions. The Pfaffl formula 2^−ΔΔCt^ [35] was used to calculate relative gene expression differences. ΔC_t_ for every mutant equals C_tMUTANT_ - C_tGAPC1_. Correspondingly, ΔC_tWT_  = C_tWT_ − C_tGAPC1_. ΔΔC_t_ was calculated as ΔC_tMUTANT_ - ΔC_tWT_.

For *35S::tso1-1 (Ler)* RT-PCR analysis (Figure S1), endogenous *TSO1* transcripts and the *tso1-1* transcripts were assayed on four individual T2 lines using *TSO1*-specific and *tso1-1* transgene-specific primers (Table S2). The PCR conditions were as follows: 95°C for 3 min, followed by 26 (or 28) cycles of 94°C for 30 s, 55°C (or 60°C for endogenous *TSO1*) for 30 s, 72°C for 60 s, and 72°C for 7 min.

### Plasmid Constructions

Using Web MicroRNA Designer, version 2 (WMD 2, http://wmd2.weigelworld.org/cgi-bin/mirnatools.pl) [36], the microRNA sequence TAATGCTGGAATAGACCGTAC that targets 3′ end of *TSO1* gene (at position 2044 bp of 2088 bp full length) was chosen to make *2044amiRTSO1*. The primers used to construct *2044amiRTSO1* were:

- mir-s: gaTAATGCTGGAATAGACCGTACtctctcttttgtattcc,
- mir-a: gaGTACGGTCTATTCCAGCATTAtcaaagagaatcaatga,
- mir*s: gaGTCCGGTCTATTCGAGCATTTtcacaggtcgtgatatg, and
- mir*a: gaAAATGCTCGAATAGACCGGACtctacatatatattcct. pRS300 plasmid was used as a DNA template. Conditions for the PCR reaction were: 95°C for 3 min, followed by 30 cycles of 94°C for 30 s, 55°C for 30 s, 72°C for 75 s, and 72°C for 7 min.

The final PCR product was first cloned into pCR8/GW/TOPO using TA cloning kit (Invitrogen, Carsbad, CA, USA) and then introduced into the pEarleyGate100 plant transformation vector [37] using the Gateway technology (Invitrogen).

For constructing *35S::TSO1-GFP*, *pAVA120* containing GFP fused to the C-terminus of TSO1 [3] was cut with PstI. The released fragment was cloned into the PstI site in the pCGN1547 binary vector.

To construct *35S::tso1-1*, total RNA was isolated from *tso1-1* inflorescences and cDNA was produced as described above. 1 µl of 10x diluted cDNA was used as a template in PCR reaction. Phusion High-Fidelity PCR kit (New England Biolabs, USA) was used for *tso1-1* cDNA amplification using gene-specific primers (Table S2). PCR conditions were as follows: 95°C for 3 min, followed by 25 cycles of 94°C for 30 s, 60°C for 30 s, 72°C for 90 s, and 72°C for 7 min. The resulting PCR fragment was cloned into pCR8/GW/TOPO (Invitrogen) and sequenced to verify the presence of the *tso1-1* mutation. The *tso1-1* cDNA was then introduced into the pEarleyGate100 binary vector [37].

For BiFC constructs, gene specific primers containing SpeI restriction sites (Table S2) were used to amplify *TSO1*, *TSO1-1*, and *SOL2* cDNAs and then cloned into pCR8/GW/TOPO (Invitrogen). After verification by sequencing, inserts were released with SpeI and cloned into the SpeI site in the pCAMBIA2300 binary vector-based BiFC vectors, pSY736 and pSY735 [38]. Genes were fused in frame and downstream of the N-terminal (YN) or the C-terminal (YC) fragment of YFP, driven by the CaMV 35S promoter, and terminated by the NOS 3′ terminator. Two negative control plasmids YC-EER5 and YN-SAC3B were obtained from Jennifer Shemansky and Caren Chang. EER5 (also named AtTHP1) was previously shown to interact with SAC3B in nuclei via BiFC [39].

### Biomolecular Fluorescence Complementation (BiFC) Assay

BiFC constructs were independently introduced into *Agrobacterium tumefaciens* strain C58C1 by electroporation. *Agrobacterium* cultures were spun down and resuspended at an OD_600_ of 0.4 in the tobacco infiltration media (10mM MgCl, 10mM MES, 100 µM Acetosyringone). *Agrobacterium* containing YN- or YC- fusion plasmids were mixed in equal parts and infiltrated into the leaves of 3–4 week old *Nicotiana benthamiana* based on a video (http://www.plantsci.cam.ac.uk/research/baulcombe/movies/agroInfil1.mpg), as well as published procedures [40]. The plants were returned to growth chamber at 25°C, 16 hr light/8 hr dark. After 48 hours, leaf sectors were placed on slides and examined under the Leica SP5X confocal laser scanning microscope with the 20x water immersion objective. YFP was visualized by excitation with an argon laser at 514 nm.

### Plant Transformation and Analysis of Transgenic Plants

Constructs were introduced into *Agrobacterium tumefaciens* GV3101 by electroporation. The corresponding *Agrobacterium* was used to transform *Arabidopsis thaliana* wild-type (L*er*) and *tso1-1* heterozygous (*tso1-1+/+sup-5*) plants via floral dip. Primary transformants were selected on soil using 1∶3000 diluted BASTA herbicide (Liberty 200). 76 *35S::tso1-1(WT)* T1 plants were generated and analyzed for phenotypic changes. Four transgenic lines were further analyzed at the T2 generation.

For *2044 amiRTSO1,* 43 T1 plants were obtained from transforming *tso1-1+/+sup-5* plants. Detailed analysis was conducted on 14 T1 plants that were confirmed to contain the transgene. Among these 14 plants, five were homozygous for *tso1-1*, four were *tso1-1* heterozygotes, and five were wild type for *TSO1* and homozygous for *sup-5*. In addition, 63 T1 plants were obtained and analyzed from *2044 amiRTSO1* transformed into wild type (L*er*) plants.

*35S::TSO1-GFP* was introduced into *tso1-1+/+sup-5* plants and 29 T1 transformants were selected on kanamycin (50 µg/ml) plates and analyzed for the presence of GFP by standard PCR. Three out of four independent lines homozygous for *tso1-1* and positive for the transgene were found to completely rescue the *tso1-1*phenotype.

## Supporting Information

Figure S1 Semi-quantitative RT-PCR showing mutant *tso1-1* and wild type *TSO1* transcript levels in four independent *35S::tso1-1 (Ler)* transgenic lines (1, 2, 3, and 4). Equal amount of total RNA, extracted from floral tissues of the four *35S::tso1-1 (Ler)* transgenic lines, was converted into cDNA, which served as templates for PCR with *tso1-1* and TSO1-specific primers (Table S2; Materials and Methods). 26 PCR cycles yielded brighter PCR bands for *tso1-1*than *TSO1* in all four lines. -RT lanes are negative controls, where reverse transcriptase was not added during cDNA synthesis to indicate a lack of genomic DNA contamination.
(TIF)

Figure S2 qRT-PCR analysis of *TSO1* transcript levels in *sol1-2; amiRTSO1* double knockdown plants and *sol1-2* single mutants. A significant reduction of *TSO1* mRNA is detected in *sol1-2; amiRTSO1* compared with *sol1-2*. Standard deviation was derived based on three technical replicates.
(TIF)

Table S1 dCAPS primers and primers used for T-DNA genotyping. To test for the presence of the wild-type allele, corresponding LP+RP primers were used, while the presence of the T-DNA insertion was tested with corresponding LB+RP primer pairs.
(DOC)

Table S2 PCR and cloning primers.
(DOC)
