# Supplementary figures and images for: Recessive Antimorphic Alleles Overcome Functionally Redundant Loci to Reveal TSO1 Function in Arabidopsis Flowers and Meristems
Source: PLoS Genet. 2011 Nov 3;7(11):e1002352. doi: 10.1371/journal.pgen.1002352 (PMC3207858; doi:10.1371/journal.pgen.1002352)

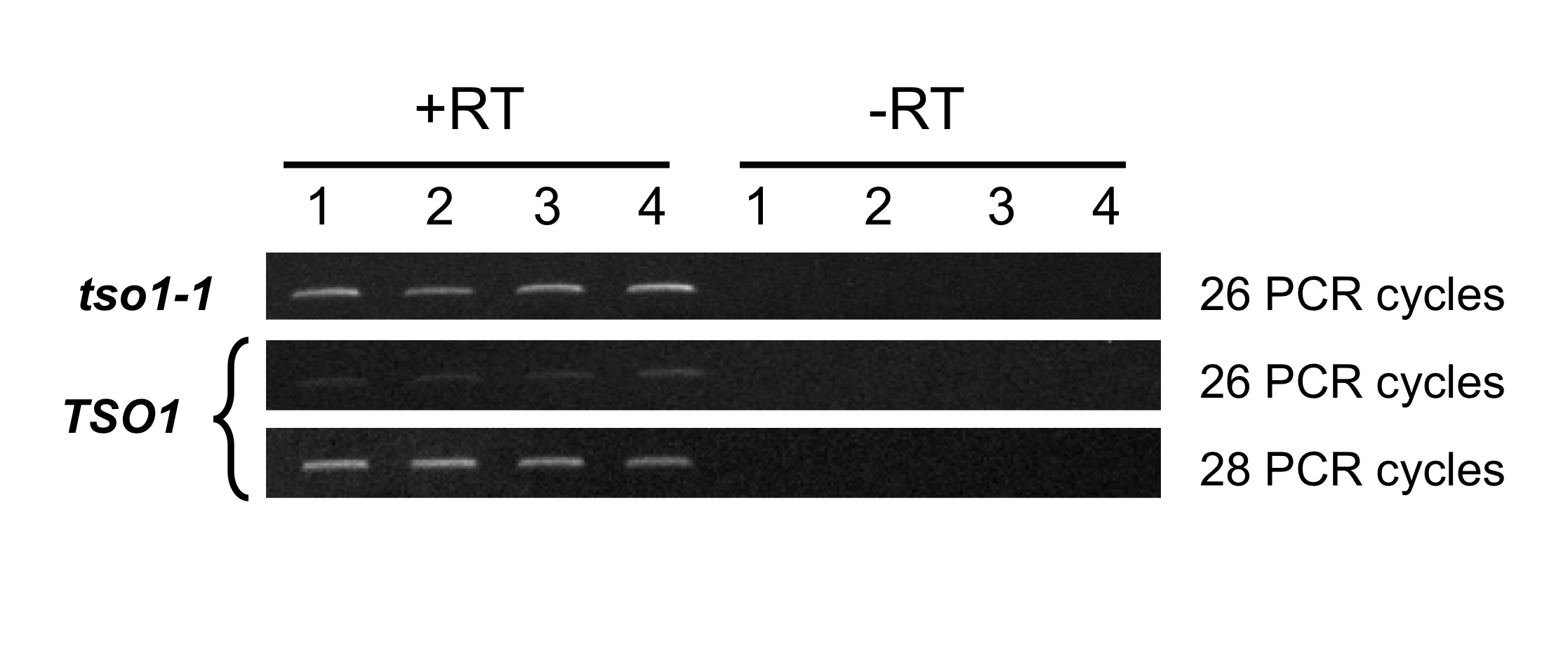

Supplement: Figure S1 — Semi-quantitative RT-PCR showing mutant tso1-1 and wild type TSO1 transcript levels in four independent 35S::tso1-1 (Ler) transgenic lines (1, 2, 3, and 4). Equal amount of total RNA, extracted from floral tissues of the four 35S::tso1-1 (Ler) transgenic lines, was converted into cDNA, which served as templates for PCR with tso1-1 and TSO1-specific primers (Table S2; Materials and Methods). 26 PCR cycles yielded brighter PCR bands for tso1-1than TSO1 in all four lines. -RT lanes are negative controls, where reverse transcriptase was not added during cDNA synthesis to indicate a lack of genomic DNA contamination. (TIF) [file pgen.1002352.s001.tif]

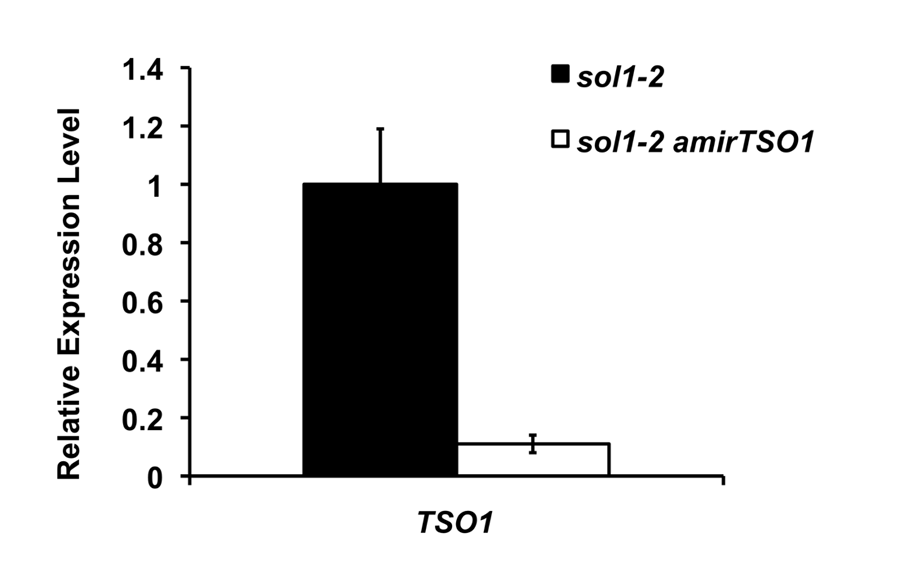

Supplement: Figure S2 — qRT-PCR analysis of TSO1 transcript levels in sol1-2; amiRTSO1 double knockdown plants and sol1-2 single mutants. A significant reduction of TSO1 mRNA is detected in sol1-2; amiRTSO1 compared with sol1-2. Standard deviation was derived based on three technical replicates. (TIF) [file pgen.1002352.s002.tif]
