# Supplementary material for: Recessive Antimorphic Alleles Overcome Functionally Redundant Loci to Reveal TSO1 Function in Arabidopsis Flowers and Meristems
Source: PLoS Genet. 2011 Nov 3;7(11):e1002352. doi: 10.1371/journal.pgen.1002352 (PMC3207858; doi:10.1371/journal.pgen.1002352)
Supplement: Table S1 — dCAPS primers and primers used for T-DNA genotyping. To test for the presence of the wild-type allele, corresponding LP+RP primers were used, while the presence of the T-DNA insertion was tested with corresponding LB+RP primer pairs. (DOC) [file pgen.1002352.s003.doc]

**Table S1. dCAPS primers and primers used for T-DNA genotyping**

| **TSO1 allele** | **dCAPS forward primer** | **dCAPS reverse primer** | **Enzyme used to cut and size of PCR product (bp)** |
| --- | --- | --- | --- |
| *tso1-1* | GCAAGAAATCAAACTGTATGAACAGATACT | GACTCCACCCTACAAGATCA | AlwNI, Mutant: 150  WT: 130 +20 |
| *tso1-3* | TGTTTTGGCTACCCGCAAACAGATTGAATCG | CCCTTAGATTAATGGAGGACAGAG | NruI, Mutant: 180  WT: 160 + 20 |
| **T-DNA lines** | LP gene primer 5’-3’ | RP gene primer 5’-3’ | **Left border (LB) T-DNA primer 5’-3’** |
| Salk 102956  *tso1-5* | TCCAATTCAGTCCCAAGACAC | CTGCTCCACTTCTTTCGACAC | ATTTTGCCGATTTCGGAAC |
| Salk 007957  *sol1-1* | AAAGGCTTGAATATCTCTGAACG | GGCTTTACACAAAACCGTACG | ATTTTGCCGATTTCGGAAC |
| Sail 742_H03  *sol1-2* | TGATTAGCAATATTCAGCCAGC | CTTTATGAGAAACCGCGTGAG | GCCTTTTCAGAAATGGATAAATAGCCTTGCTTCC |
| Sail 78_A12  *sol2-1* | TCCTCCTCCTTCTTCTTCCAC | TCAACTGAAGCTTCTTCTCGC | GCCTTTTCAGAAATGGATAAATAGCCTTGCTTCC |
| Salk 021952  *sol2-2* | AGATTGCAGACAAAGCAAAGC | TGGAGAATCCTGCATTTTCAG | ATTTTGCCGATTTCGGAAC |
