# Supplementary material for: Recessive Antimorphic Alleles Overcome Functionally Redundant Loci to Reveal TSO1 Function in Arabidopsis Flowers and Meristems
Source: PLoS Genet. 2011 Nov 3;7(11):e1002352. doi: 10.1371/journal.pgen.1002352 (PMC3207858; doi:10.1371/journal.pgen.1002352)
Supplement: Table S2 — PCR and cloning primers. (DOC) [file pgen.1002352.s004.doc]

**Table S2. PCR and cloning primers**

| **Real time RT-PCR primers and primer efficiencies** | | | |
| --- | --- | --- | --- |
| *Gene name* | *Forward Primer* | *Reverse Primer* | *Efficiency (%)* |
| *TSO1* (At3g22780) | CACTGCCACCGTACAGACATTTG | AAAGAGGAAGAACCTGTGCCAAGA | 110 |
| *SOL1* (At3g22760) | CTCAAAGTCGAGGATTCACCAGT | GAAAACAGAAGGTGGAGATGCCT | 107 |
| *SOL2* (At4g14770) | CCGGAAAGATGGGTCTTCCATTG | TCGTAGGTAGAGCAGAACTCATGTCC | 100 |
| *GAPC1* (At3g04120) | CCAGTCACTGTTTTCGGCATCA | AGCTGCAGCCTTGTCTTTGTCA | 98 |
| **RT-PCR primers** | | | |
| *Gene name* | *Forward Primer* | *Reverse Primer* | *Purpose* |
| *tso1-1 transgene* | TGTGAATGCTATCAGGGTGGAGTC | AGAAAGCTGGGTCGAATTCGC | Fig. S1 |
| *TSO1* | TGTGAATGCTATCAGGGTGGAGTC | GATAGGCTAATAGGATCTGGAAC | Fig. S1 |
| **Cloning primers** | | | |
| *Gene name* | *Forward Primer* | *Reverse Primer* | *Purpose* |
| *tso1-1* | ATGGACAAATCCCAGAAGAATCCT | TCACTGATTTGGGTTGAGAGAAGG | *35S::tso1-1* |
| *TSO1* | CTCACT AGTATGGACAAATCCCAGAAG | CTGACTAGTCACTGATTTGGGTTGAGA | BiFC constructs |
| *tso1-1* | CTCACT AGTATGGACAAATCCCAGAAG | CTGACTAGTCACTGATTTGGGTTGAGA | BiFC constructs |
| *SOL2* | ATACTAGTATGGATACACCGGAAAAGAGT | ATACTAGTTTAATGGTGTGGAGTGAGAGA | BiFC constructs |
